# Modeling and inference of mixed dynamics and detection of causal emergent features

**DOI:** 10.1038/s41598-025-29523-z

**Published:** 2026-01-14

**Authors:** William Casey, Leigh Metcalf, Shirshendu Chatterjee, Heeralal Janwa, Ernest Battifarano, April Edwards, Yaron Gvili

**Affiliations:** 1 United States Naval Academy, Annapolis, MD US; 2 Carnegie Mellon University, Pittsburgh, PA US; 3 City University of New York, Graduate Center and City College, New York, NY US; 4 University Puerto Rico, Río Piedras, San Juan, PR US; 5 New York University, New York, NY US; 6 Independent Scholar, Tel Aviv, Israel

**Keywords:** Mixture model, Change-point analysis, Nonlinear least squares, Epidemiology, Logistic equation, Time series modeling, Forecasting, Causal inference, Mathematics and computing, Applied mathematics, Computational science, Statistics, Statistical methods

## Abstract

Many real-world problems feature nonlinear dynamic processes. Classical mathematical models may be adequate to describe a single dynamic process in isolation, but can be easily undermined by two natural and simple kinds of phenomenological variations: the emergence (or activation) of an additional dynamic process, and events that affect the parameters of an active process. COVID-19 data offers an important case study expressing these phenomenological variations that deeply challenge the classical SIR epidemiological model, and call for novel mathematical methods to detect and adapt to these critical variations. We address the modeling issues with a novel mathematical framework that reenvisions data as a mixture of multiple causal generating processes, each subject to possible parameter change-points. The new viewpoint extends nonlinear classical models in a manner that overcomes many of these types of phenomenological variations and enables a highly adaptive modeling closely linked to causal events. The new model space unifies a wider class of dynamics and is particularly effective at fitting multi-surge data and explaining key causal events related to surge origination. To demonstrate, we construct a mixture of logistic models termed the Adaptive Logistic Model (ALM), and then formulate appropriate nonlinear least squares optimization and regularization goals, and then apply ALM to data. To validate the approach, we return to COVID-19 forecasting (for case count), and compare ALM directly to other forecasting methods. ALM forecast accuracy is competitive with all leading forecast methods, but its greatest utility may be in how it detects changing dynamics (change-points) and retains far fewer but more interpretable parameters relating naturally to cause and intervening change. The method can be applied more generally as it adapts well to the multi-generative nature of many time series data problems. We demonstrate ALM robustness through data experiments in hydrology, economics, cybersecurity, and social media.

## Introduction

The infection dynamics of SARS-CoV-2 has proved challenging for mathematical modelers; nevertheless, mathematical models that offer reliable, repeatable predictions are essential for informed policy that can reduce human harm. The pandemic has highlighted multiple limitations of standard mathematical modeling tools^1^, such as the use of the classical compartmental SIR model to predict case loads. As the pandemic unfolded and more data became available, the limitations of classic models became more evident. Modelers identified a variety of novel factors including^2–4^: vaccination distribution^5,6^, super-spreaders^7^, policy changes^8^, behavior changes^9^, economic impacts^10^, vaccination hesitancy, and misinformation or counterproductive narratives^11^ are only some of the novel considerations that needed to be incorporated to improve infection case load prediction models.

Pandemic modeling has also motivated novel advances and enhancements of standard mathematical models: the standard SIR models have been enhanced to account for multi-surge events in^7^; multilayer network models overlays for epidemiology are considered in^12^; and self-exciting branching models are considered in^1^. Those models discover similar phenomenological variations, and, like our method, are similarly motivated to address the shortcomings of standard models. Our approach, demonstrated with ALM, is to unify a wider class of models remixing classical ones, the new class can capture much more complex phenomena than an SIR model. Our contributions are 1) identifying phenomenological variations which challenge classical models, 2) developing a framework for a mixture model that addresses multi-causal dynamic processes, and 3) connecting inference, forecasting, causal events, change-point detection, and model adaptation with objective optimization within the framework.

**Phenomenological variations:** We argue that the inherent difficulties of COVID-19 modeling in the last few years stem from at least three kinds of adaptations; we refer to the list below as *phenomenological variations*. *First,* that multiple dynamic conditions can arise or be activated. COVID-19 case loads were the result of multiple simultaneously active infection processes caused by distinct viral variants. Viral variants were distinct enough to reinfect individuals, suggesting that the infection processes act (at least partially) independently. Additionally, new strains arise from mutation, introducing time delays between the infection processes as well as differing infection rates. Random events (e.g., the arrival of a new strain) that significantly alter the course of evolution are incorporated as change-points. Although these random events are initially unobserved, our procedure can estimate the change-points and subsequent dynamics reasonably well and proficiently (meaning the new trend can be identified quickly after the change). Critically we argue that case load numbers are an aggregation or mixture of case load numbers arising from separate strains. Multi-surge models^7^ have also addressed the need for multiple independent dynamic processes. *Second*, that changing parameters of existing processes, policies, and behaviors have been aimed directly at modulating infection dynamics (e.g. *flatten the curve*). These effects may include vaccination distributions or behavioral changes in response to viral dynamics (i.e., isolating when infection rates increase). And *third,* and perhaps most difficult, is that novel nonlinear dynamic conditions can still emerge. Early efforts to model COVID-19 case loads, witnessed much speculation about novel nonlinear dynamics with various casual possibilities: silent-carriers, super-spreaders, behaviors such as super-spreader events. Our method is designed to address the first two issues with flexible model frameworks that can achieve model accuracy even with these phenomenological variations.

The inherent difficulties above are neither isolated to infection dynamics, nor less appreciated in other scientific fields. We sense that there are many applications. In particular, the application of virology to economics^13^ and other social sciences are ready examples. Other examples from earth science and biology include hydrology, geology, climate change. We illustrate our method on problems from diverse disciplines which also importantly require policy tools.

## Background

To address the demands of modeling case load data, we have designed a method that considers mixtures of dynamic processes defined by ordinary differential equations. Our method is further structured to detect and self-adapt to changing parametric regimes. Our method draws upon mathematical aspects from several topical areas: mixture modeling, time series (temporal data) analysis, change-point detection, dynamical system inference and learning, nonlinear least squares, and infection models. To our knowledge (and surprise), no prior work has integrated these aspects into tools capable of addressing the demands of the applications examined in this work.

Mixture models have been explored in various mathematical and data science models that considered parameter estimation for mixture models. The Gaussian Mixture Model and associated Expectation Maximization (EM) algorithm^14^ considers mixture data of two Gaussian distributions. Radial Basis Boltzmann machine^15^ extends the theory to a larger number of Gaussian distributions. Mixture models have also been considered for temporal data^16–21^ however the aspect of identifying and adapting to change-points is unclear.

Time series analysis has a history of foundational work and many data-oriented methodologies^22^. Within this domain, Bayesian forecasting models^23^ have been extensively explored, including approaches for change-point detection^24^–especially in scenarios where the goal is to identify departures from a static distribution. Another major line of research emphasizes frequency-domain techniques and detrended time series models such as ARIMA^25^. However, these traditional methods often struggle to accommodate changes in underlying trends^26^ and can be computationally expensive^27^— limitations that our model effectively addresses.

Numerous change-point detection methods have been proposed: Binary Segmentation^28^, Bayesian models^24^, and Total Variation Minimization^29^. Other approaches include^30–35^. Dynamic processes with changing parameters have also been considered with Non-Homogeneous Poisson Processes^36,37^; however, these provide no immediate means to scale beyond one or two change-points. Another model along similar lines is overdispersion in Poisson processes^38^ that has features of an adaptive model.

Although some approaches integrate time series analysis with change-point detection^24^—closely resembling our own optimization strategy—to our knowledge, existing change-point methods have not addressed mixture models of dynamic equations, nor have they considered even the relatively simple case of growth or decay ODE models. Consequently, theoretical results in this area remain limited. To address these gaps, we provide the mathematical foundations of the ALM model in the Appendix, while reserving a deeper investigation of the model’s robustness for future work.

Dynamical inference models have included system identification^39^ and, more recently, physics-inspired deep learning^40–42^. Nonlinear least squares estimation problems have considered temporal data and more generally linear separability^43^ and combination of nonlinear basis functions in the variable projection method^44^, which we make use of in this work. These techniques are inherently suited for mixture models, however, application to model growth and decay processes has been underdeveloped until now.

Our method was motivated by and developed to model multiple surges of SARS-CoV-2 case numbers with greater accuracy than compartmental SIR models. The SIR model is the classical mathematical model for epidemiology, while appropriate for a single pathogen affecting an isolated and simple population^45,46^, it ultimately proved less useful for modeling SARS-CoV-2 spread within the human population. At the outset of COVID-19, the SIR model provided a reasonable approximation for case count; however, as the second wave of cases began, its validity was undermined by a widening discrepancy between model prediction and actual case count data. Unfortunately, as new variants emerged^47^ that reinfected people who had been fully vaccinated for protection against earlier variants, the SIR model was shown to fail in these global circumstances^48^. Also, at the beginning of the pandemic, an attempt was made to fit the logistic model to the cases^49^. However, it was shown that in the absence of re-parameterization, it is a poor fit for the emerging surges. We believe our modification of the model as discussed below solves this problem effectively.

Our method is next demonstrated on the logistic nonlinear ordinary differential equation, which can model both growth and decay processes characterized by two parameters: capacity and rate.

## Materials and methods

We begin with the logistic equation, a nonlinear ordinary differential equation (ODE) whose solution is the core nonlinear function we use to demonstrate our methodology. Next we consider the problem of parameter estimation. We expand the model using a class of affine combinations over the model class, enabling successful modeling of multiple nonlinear surges of the same mathematical nature. We readdress the estimation problem and provide a regularized objective that, while preventing overfitting, can be designed to detect change-points. We detail the numerical methods enabling fast convergence for model estimation; we discuss the adaptive capability of the model and illustrate its connection to change-point detection and how the model adapts to novel changes in data.

### Nonlinear dynamics: the logistic model

The origin of the logistic model dates to the late 18th and early 19th century. At that time, mathematical and philosophical discussions on the stability of growth were common; one such economic example posed by Malthus is whether abundance should be used to further growth or invested in enhancements toward a utopian society. Mathematical models describing growth available at that time included *arithmetic* and *geometric* growth now respectively termed linear and exponential [Geometric models include a wide class of solutions to difference equations with constant coefficients such as the Fibonacci recurrence $F(n) = F(n-1) + F(n-2)$, whose closed bounded solution: $F(n) = \frac{\phi ^n - {\left( 1 -\phi \right) }^n }{\sqrt{5}}$ combines two geometric growth modes]. These models generally struggled to capture both accelerating growth and any reasonable counterbalancing dynamics to yield an equilibrium. Pierrer-François Verhulst working with Alphonse Quetelet adapted the exponential growth model to include a term with sufficient resistance to limit growth, thus capable of obtaining an equilibrium. During the course of a three-paper sequence in 1838-1847, the logistic model emerged^50^.

Today the logistic model is widely known in its ordinary differential equation (ODE) form, letting *X*(*t*) representing mass at time *t*:

M1 $$\begin{aligned} \frac{dX}{dt} = \underbrace{r X(t) }_{growth} \underbrace{ \left( 1 - {X(t)}/{K} \right) }_{resistance}, \end{aligned}$$

and owing to its elegance and expressivity, its applications are numerous and diverse. It is widely applied in many fields: biology, ecology, physics, chemistry, material sciences, electrical engineering, and machine learning where the sigmoid function is an important neural network activation function.

Logistic growth M1 is a first-order nonlinear ordinary differential equation, having two parameters: capacity *K*, and rate *r*. With $r<0$ the dynamics can also express capacity-limited decay. Solutions for M1 with initial conditions $(t_0, X_0)$ exist and describe capacity-limited growth or decay under reasonable assumptions for $X_0$ and *K* listed in Table 1.

The equation M1 with initial conditions (IC) of $X_0$ at time 0 has a closed-form solution:

S1 $$\begin{aligned} X(t) = {\left\{ \begin{array}{ll} \frac{ X_0 K }{ \left( K - X_0 \right) e^{ -r (t -t_0) } + X_0 } \text { for } t>= t_0 \\ 0 \text { otherwise when } t < t_0. \end{array}\right. } \end{aligned}$$

For $t>t_0$ the solution coincides with the sigmoid function (or the hyperbolic tangent function), a smooth infinitely differentiable function whose domain is the real line:

S2 $$\begin{aligned} f(t ; r, K , t_0, X_0 ) = \frac{ X_0 K }{ \left( K - X_0 \right) e^{ -r (t -t_0)} + X_0 }. \end{aligned}$$

Slightly different from considering governing dynamics (equation M1) and initial conditions, is to consider a class of solution curves (equation S2) and one observed point on the solution curve. For the logistic model, this alternate framing uniquely determines the same solution, and is particularly useful if the initial conditions are not observed – such as the case of parameter estimation from data.

**Table 1:** Behavior characterization for the solution S1. The logistic equation is capable of expressing capacity-limited growth, and capacity-limited decay, as well as a constant (nonchanging) dynamics. The constraints and asymptotic limits are shown for each case.

**Behavior**	*r*	*K*	$\lim _{t \rightarrow \infty } X(t)$
Capacity-limited growth	$r>0$	$K> X_0$	*K*
Constant	$r=0$ or $K = X_0$		$X_0$
Capacity-limited decay	$r< 0$	$X_0>K$	*K*

The solution S1 and sigmoid function S2 match identically for $t>t_0$, and are capable of expressing either growth or decay, as well as the desired properties, sought by Verhulst, initially explosive then increasingly resistive resulting in frozen equilibrium at capacity.

Additional properties of the mathematical model and derived formulae for the logistic equation can be found in the Appendix. We summarize the main derivations needed.

First, the inflection point of the function *X*(*t*) in equation S1, is the point at which growth is momentarily matched by resistance to growth. It is given by:

S3 $$\begin{aligned} \tau = \frac{\log { \left( \frac{ K - X_0 }{ X_0 } \right) }}{r}. \end{aligned}$$

Second, using the inflection point $\tau$, we can develop formulae to relate the amount of change in the equation S2 to a time interval symmetric about $\tau$. For equation S2, the time when any fraction (say *q*) of total change (*K*) occurs can be calculated by solving $X(t) = q K$ for *t*, the crossing time is given by:

S4 $$\begin{aligned} t_q = \tau - \frac{ \log {\left( \frac{q}{1-q}\right) }}{r}. \end{aligned}$$

These formulae are useful for interpreting dynamics. For example, when parameters are fixed they allow us to determine a point in time $t_q$ when a fraction *q* of the total dynamic change for *X*(*t*) will be observed as historical. Noting the symmetry in *q* around 1/2, we can derive a formula that determines double sided time-bounds for which a given amount of dynamic change occurs (see appendix). Additionally, since the solution for S1 and equation S2 only differ for $t< t_0$, the formula can also be used to measure the difference between an ODE solution with initial conditions and our alternate framing. Further explanations are given in the appendix.

*Summary:* Not every nonlinear model admits a closed-form solution, but the logistic equation yields helpful and elegant formulae when considering prediction and adaptive aspects of the full model. Still, the logistic function is expressive enough for a host of interesting capacity/rate limited dynamics. For nonlinear ODEs without analytic solutions, numerical techniques can augment approaches along these lines, albeit much more computationally intense augmentations of methodology are required. We continue the methodology assuming the logistic formula as our nonlinear model.

### Logistic model estimation

Given data for a growth/decay process governed by the logistic equation M1, one may ask which curve (S2) fits the data best.

Letting $D = \langle (t_i, d_i) \rangle _{i=1}^N$ be data, the squared residual can be defined by:

$$R( r, K, t_0,X_0 | D ) = \sum _{i = 1}^N \left( f(t_i ; r, K, t_0, X_0 ) - d_i \right) ^2.$$

Minimizing $R(r,K, t_0,X_0 |D )$ can be carried out using numerical methods, a technique known as nonlinear least squares regression.

In this setting, the variables $t_0, X_0$ can be interpreted as any point of the curve. Letting $\theta = \langle r, k, t_0, X_0 \rangle$, Model estimation is a search extending over the admissible domain ${\mathscr {X}}$ to determine a minimizer:

$$\hat{\theta }= \underset{ \theta \in {\mathscr {X}}}{\arg \min } R( \theta | D ).$$

More generally, the model estimation procedure can be further refined and tailored if one knows the error distribution.

### The affine model

To provide some modeling resolution for COVID-19’s multiple waves, we use a linear sum of these nonlinear components. We term this additive model composed of logistic components the *Adaptive Logistic Mixture Model* or **ALM Model**. Let ${\mathscr {M}}$ be the class of all curves from equation S2. Letting $\theta _i = \langle r_i, K_i, t_{0i}, X_{0i} \rangle$, we may account for various time conditions of the causal effect for a set of independent surges indexed by *i*. A discussion of the admissible set of parameters $\theta$ can be found in the mathematical model portion of the supplemental materials. We use ${\mathscr {P}}$ here.

Let

M1 $$\begin{aligned} {\mathscr {M}}^N = \{ \sum _{i=1}^N f(t ; \theta _i ) | \theta _i \in {\mathscr {P}} \}, \end{aligned}$$

and let:

$${\mathscr {M}}^+ = \bigcup _{J = 1 }^\infty {\mathscr {M}}^J,$$

that is, the set of all finite sums over members of ${\mathscr {M}}$.

#### Nonlinear least squares with regularization

We generalize a procedure for fitting data by residual formulae $R_N$ applied to curves in the class ${\mathscr {M}}^N$. Next, we describe a regularization term *C* that acts to select the number of components required, penalizing the over-utilization of additional logistic components. Together, $R_N$ and *C* provide a residual function $R_*$ for the class ${\mathscr {M}}^+$, thereby defining the model estimation objective for the additive model.

Letting $D = \langle (t_i, d_i) \rangle _{i=1}^n$ be data, the squared residual displacement from the data to a curve of ${\mathscr {M}}^N$ specified by $\langle \theta _1, \cdots , \theta _N \rangle$ can be defined as:

R1 $$\begin{aligned} R_N ( \theta _1, \cdots \theta _N | D ) = \sum _{j=1}^n \left( \left( \sum _{i = 1}^N f( t_j | \theta _i) \right) - d_j \right) ^2 . \end{aligned}$$

A nonlinear least squares procedure can determine efficiently local numerical minimizers on ${\mathscr {M}}^N$ given data. Let $\Theta = \langle \theta _1, \theta _2, \cdots , \theta _N \rangle$, ranging over admissible value set ${\mathscr {P}}^N$, then:

R2 $$\begin{aligned} \hat{\Theta }(N) = \underset{ \Theta \in {\mathscr {P}}^N }{\arg \min } R_N ( \Theta | D). \end{aligned}$$

Letting ${\mathscr {P}}^+ = \cup _{j=1}^\infty P^j$, a minimizer of ${\mathscr {M}}^+$ can be defined with an appropriately chosen cost function. For $\Theta \in {\mathscr {P}}^+$ let $N(\Theta )$ measure the number of parameter indices. Finally, the regularized residual is:

R3 $$\begin{aligned} R_*( \Theta | D ) = R_{N(\Theta )} ( \Theta | D ) + C( N(\Theta )), \end{aligned}$$

where *C* is a monotonically increasing cost function, is designed to penalize the use of additional parameters for curve estimation. The minimizer is defined by:

R4 $$\begin{aligned} \hat{\Theta }= \underset{ \Theta \in {\mathscr {P}}^+}{\arg \min } R_*( \Theta | D). \end{aligned}$$

### Change prediction and auto-adaptive modeling

Here we describe one of the model’s key features: its ability to recognize when a new dynamical regime is emerging and to adapt quickly with new parameters. A new parametric regime or a change-point imputing significant changes to existing parameters can be detected in our model in a variety of ways including: statistical testing, hypothesis testing, and optimized dynamical programming.

**Fig. 1:**
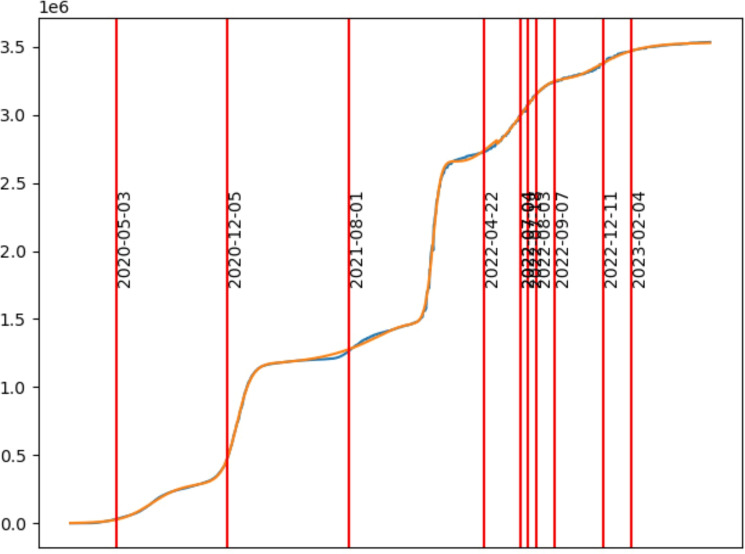
COVID-19 case (cumulative) count dynamics in Los Angeles County 2020-02-02 through 2023-07-23 illustrates the basic modeling problem. Cumulative case counts (blue) arise from several waves of growth. Our model, called ALM, considers affine mixtures of multiple nonlinear dynamic models. By extending classical models in this way, data can drive a nonlinear regularized least squares optimization to achieve better models (orange) whose parameters indicate important changes points (red vertical lines). For COVID-19 case loads, the model determines change-points consistent with surge events. Importantly, ALM provides a means to model multi-surge growth in ways that compartmental models are not suited for.

During the COVID-19 epidemic, novel strains of the SARS-CoV-2 virus emerged to reinfect individuals who had recovered from earlier strains, thereby generating multiple waves (or surges, for example, see Fig. 1). Nonetheless, the effect on cumulative case counts appears to be driven by the aggregation of two independent growth components each with its own parameters. For capacity/rate dynamic evolution such as the other examples below, and as described by the affine model with *N* surges (equation M1), the model, owing to its regularization term, is equipped to determine when an additional growth component should be included. This can happen when

D1 $$\begin{aligned} \frac{ | R_N ( \hat{\Theta }(N) | D ) - R_{N+1} ( \hat{\Theta }({N+1}) | D ) |}{ C(N+1) - C(N)} < 1. \end{aligned}$$

Said differently, additional parameters, stepping from a model in ${\mathscr {M}^N}$ to a model in ${\mathscr {M}}^{N+1},$ is worth it when the reduction in residual outweighs the cost. As such, these events occur as jump processes in $N(\Theta )$ with respect to equation R3.

More generally, we can adopt the view that *distinct causes* can arise at any point, resulting in the addition of new dynamic components that are governed by the same dynamic laws.

**Change Detection:** We propose two methods to enable the model to self-identify the emergence of novel dynamic components and consider the decision criteria of equation D1 to trigger the use of additional parameters. Also important for policy and prediction is the time required to detect change, as well as the time required for parameters to stabilize after the change is detected. First, by monitoring the skewness for residual fit, the models can determine the plausibility of evidence of a recent change. Letting $r_k = \left( \sum _{i = 1}^N f( t_k | \theta _i) \right) - d_k$. A skewness test can be applied to $r_k$, under the assumption that $\langle r_k \rangle _k$ follow a normal distribution with mean $\bar{r}$ and standard deviation $\sigma$ for data *r*[: *m*]:

D2 $$\begin{aligned} \mu _3(m) = \frac{ \sum _{k=1}^m ( r_k - \bar{r} )^3 }{ \left( m - 1 \right) \sigma ^3 }. \end{aligned}$$

A threshold can be set once $| \mu _3(m) |> \tau$, and a wider search for extended parameters can be triggered.

Second, by direct model hypothesis testing, letting *D*[..*k*] be the observed data up to and including time step *k*, we extend equation R4 to account for a number of observations. Let

D3 $$\begin{aligned} \hat{\Theta }_k = \underset{ \Theta \in {\mathscr {P}}^+}{\arg \min } R_*( \Theta | D[..k]), \end{aligned}$$

and let ${\mathscr {J}}_k= N( \hat{\Theta }_k )$ describe the number of parameters best describing data at time *k*.

At time $k-1$, assume ${\mathscr {J}}_{k-1} = J$, when data sample for time *k* is observed, perform modeling in both classes, ${{\mathscr {M}}^J}$ and ${\mathscr {M}}^{J+1}$, then by directly applying the decision criteria of equation D1, the model can self-evaluate if more parameters are required. In this way, the model is constantly considering hypothesis testing in two classes of models, ${\mathscr {M}}^J$ the current model, and ${\mathscr {M}}^{J+1}$ which can improve model fits under the assumption that a novel dynamic component has recently emerged.

Additionally, there are variations. Hypothesis testing can be expanded to consider many classes, or more efficiently, one can use the skewness test to trigger hypothesis testing.

The ability of the model to detect change given recent observations, is consistent with other change-point methods, such as online Bayesian change-point detection^24^; however, note that our method requires no prior distributions for parameters. The ability to self tune fills a gap noted in^49^, where the need for new parameters was recognized. Our method detects change and determines new parameters.

### Numerical algorithms for multi-causal model

The nonlinear least squares problem is most commonly addressed and solved ’locally’ with the Levenberg-Marquardt method^51,52^. Given an initial setting for parameters, a search in parameter space, guided by the objective of decreasing the residual fit, will determine a minimizer (at least locally in the vicinity of the initial parameters). Quicker convergence is offered by various enhancing search strategies: the Trust Region Reflective (TRR) algorithm^53^ offers improved handling of over/under shooting parameters bounds, and the Variable Projection method^44^ method decomposes the search into two sub-searches: parameters affecting nonlinear kernels and parameters which combine nonlinear kernels into an affine model. Our code, available as open source (see appendix), leverages each of these methods via the Levenberg-Marquardt solvers curve_fit within the Python modular library scipy.optimize^54^, and the Variable Projection library within the VarPro Rust language library^44^.

Our code, leveraging the efficiencies of the Levenberg-Marquardt solvers, can infer model parameters rather quickly. We implement the nonlinear least squares curve-fitting inductively. Starting with a single logistic, we determine $\phi _1$, then building inductively we determine $\phi _{n}$ by holding all prior estimated parameters (i.e., $\phi _1, \phi _2 ... \phi _{n-1}$) fixed, and apply the solver to minimize $R_n$ defined in equation R1. The routine can be stopped when the residual improvements fall below a specified threshold.

Running on a dual-core MacBook Pro (Version Ventura 13.4.1) with 32G of memory, our code implementing reflection solvers from scipy.optimize, can determine the parameters for a two surge model given 500 data points in an average 2.5 seconds. When we increase the data set size to 1500 points, curve-fitting can be achieved in an average 3.5 seconds. Our code which implements the VarPro method is even faster (see nonlinear least squares in the appendix)

## Results

We first illustrate the outcome of nonlinear least squares regression for ALM on COVID-19 data in Fig. 1. We conclude from its residual, that ALM models effectively COVID-19 case load in ways that other approaches, such as compartmental SIR models, struggle with. Further, we compare ALM to more than 30 statistical forecasting methods (additional materials on COVID-19 forecasting are found in the appendix) by directly comparing Least Squared Error (LSE) of forecasting to data in a retro-diction study. There we find that ALM with little configuration, is already a notable competitor among forecasting methods, even with 21 or fewer parameters to develop an entire baseline model for over three years of forecasting. An additional feature of ALM includes the elucidation of inflection points. These aspects enhance the explainability of the model and can potentially enhance policymakers reasoning about diverse causes including emergence of novel strains as well as interventions such as policy shifts. ALM achieves outstanding model fit and explainable summary to include surge inflections that feature multiple growth phenomena as time localized surges.

Next, we consider, more broadly, applications of ALM by examining model related problems in various science fields where capacity/rate parameterized growth/decay components are episodically active due to potentially hidden causes. Further, we discuss ALM’s potential enhancing use in those problem domains. We discuss change prediction and what insights change prediction can offer for policymakers, in particular by detecting emergent and novel dynamics as they unfold, timely reasoning and decision-making can be applied. The logistic model, and its historical resolution of capacity-limited growth, has been a powerful general model for dynamics in many areas of science. We believe that this data-driven extension, called ALM, may also help capture essential features in a wide range of scientific problems that feature confounding aggregations of multiple components of capacity-limited growth and decay, including biology, epidemiology, earth science, hydrology, economics, cybersecurity, and social media. We conclude this results section with a discussion of model limitations.

### Biology and epidemiology

We apply the ALM model for prediction of case loads due to SARS-CoV-2 during the COVID-19 pandemic. Better prediction of case loads is important for planing and policy-making and can save lives. In Fig. 1, ALM is applied to cases from Los Angeles County. Additionally, to compare with state of the art statistical methods, we provide a comprehensive comparison study of methods for COVID-19 case count prediction tasks. The study limits the scope to the state of California over more than three years of time and compares only to methods that offered more than 80 predictions in Covid-Forecast hub. See the appendix for comparison and results.

### General applications

Here, we consider and illustrate the use of the additive logistic model ALM to various scientific subjects.

**Fig. 2:**
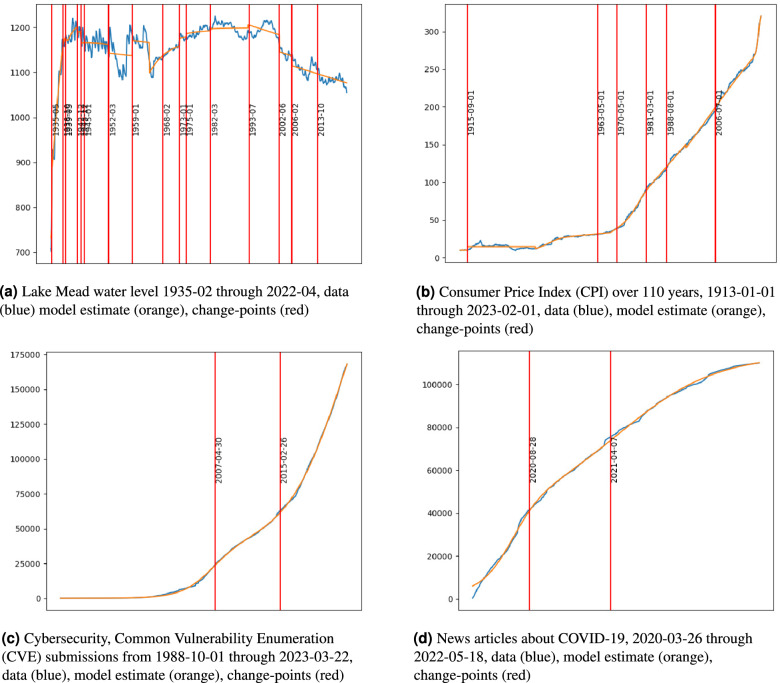
Applications of the ALM model to various dynamic processes that are challenging to model with a single isolated classical model. By extending the hypothesis space to a mixture of multiple repeated models, the classical model repertoire can be used as a basis to assemble well fit models that better reflect data and identify changing dynamic conditions. The ALM model which considers a mixture of logistic growth and decay modes can generally fit a wide range of problems involving rate and capacity-limited growth or decay.

**Earth science and hydrology**, such as water levels in reservoirs (for example Lake Mead), have extrinsic and intervention causal components which modulate capacity and rate for water levels as a function of time. Causes include drought, changes in demand, and policy^55^. Figure 2a illustrates ALM on Lake Mead water levels.

**Economics**, such as the Consumer Price Index (CPI), has been studied since 1913 and is used in determining inflation^56^. Figure 2b illustrates the applications of ALM to CPI. The number, published monthly, is essentially the rate of change of the data plotted in 2b. Additionally, the possible use of Narrative Economics^13^, features an overlap of applications of economics with social media. Analogously to the famed butterfly effect in weather prediction, it would be interesting and perhaps useful to quantify how talking about the economy changes the economy.

**Cybersecurity**, vulnerabilities are important information regarding code bugs that hackers can exploit. As such, they represent an informational commodity for cyber operations and are tracked by the US National Institute of Standards and Technology (NIST) within their National Vulnerability Database (NVD). By considering the frequency and date of incident report associated to each vulnerability, a type of epidemiology can be developed to track cybersecurity. Accordingly, the ALM model also performs well within this domain as shown in Fig. 2c.

**Social media**. The rise of social media has enabled novel modes of information sharing, and raised new concerns of viral miss-information. We apply the ALM model to counts of news articles re-posted per day that mentioned the COVID-19 pandemic, we present ALM modeling in Fig. 2d.

#### Predicting change and auto-adaptive models

**Fig. 3:**
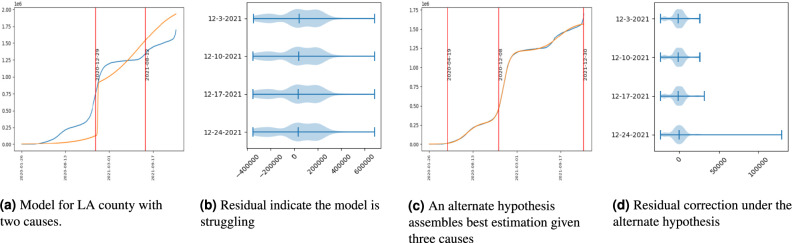
Forecasting with the ALM model: One advantage of this approach is that the model is able to self-evaluate its residual fit on a running basis. Should the residual, assumed to be Normal, accumulate sufficient skewness, the model can form a model selection test to include a hypothesis that a change-point has recently occurred. By comparing the current model (e.g., in (**a**) having two causes for growth), to an alternate model which posits an additional generating process (e.g., in (**b**) a third cause is introduced). Change to the model is accepted if the model improvements (correction for skewness or to improve the residual) outweigh the cost for adding an additional component imposed by the regularization term. This can be efficiently automated and performed by the model on a recurring basis. New dynamic regimes can be identified shortly after evidence mounts that data diverges from the current best forecast model.

The ALM model responds to and auto-tunes new parameters to emergent dynamic components (surges) found in data from LA county COVID-19 cases for various time periods (i.e., using *D*[..*k*] to represent the first *k* samples of data as would be observable on day *k*). We illustrate how the model in classes ${\mathscr {M}}^2$ and ${\mathscr {M}}^3$ adapts to minimize residual shortly after observing data from the third wave of cases. In Fig. 3 the considerations used by the model to change and re-trigger estimation with additional parameters are visible. In Fig. 3a we observe that at around 2021-08-12 the fit for a two surge model begins to struggle, in 3b the residual distributions for *r*[..*k*] are visualized for weekly updated data (i.e., for *k* equal to 2021-12-03, 2021-12-10, 2021-12-17, and 2021-12-24). In contrast, model estimation with three surges (additional parameters) are found to fit better in Fig. 3c, reduce residuals and restore symmetry in residual distributions (see the violin plots of Fig. 3c displaying the residual for ${\mathscr {M}}^3$ for same dates).

Accordingly, the loss of symmetry for two surge models can be seen in the distributions of Fig. 3b. Further, the loss of symmetry is detectable with a skewness measure. See equation D2. Also, residual reduction can be detected within the outlined hypothesis testing and decision criteria of equation D1. Note that the decisions to switch from a model in ${\mathscr {M}}^2$ to a model in ${\mathscr {M}}^3$ can be made fairly early on, as early as 2021-08-12 as the beginning of the third wave is experienced.

#### Explainable models and interpretations

Fitted models are amenable to interpretation. First, our software can decompose the components of growth. Note that the inflection point is highlighted as red vertical lines in Figs. 1, 2a, b, c, and d to represent a specific time centering the dynamics of a single additive component. Additionally, the inflection point is where the growth rate for this component is optimal. Using equation S4, the inflection point can be used to locate a time when a percentage of change (due to the parameters of the given component) has occurred. Our software locates the positions of these inflection points and relevant bands around them and finds the associated capacity and rate parameters, where any portion of its effect is observed, as well as component parameters. This information is intended to enable policy formation and assessment tasks, where explainable AI algorithms are preferred to black-box methods that struggle to reveal what features of data are critical to the optimization results.

#### Limitations

While the additive logistic model (ALM) performs well on various data sets that feature temporally localized growth or decay modes, its effectiveness may be limited by both noise and the possibility of temporally overlapping growth/decay epochs that confound model estimation.

The design of the cost function found in equation R3 is an important and interesting problem associated with change-point detection and identification. Here, we argue that many problems are amenable to a design approach where domain experts, knowledgeable of what constitutes change, could develop appropriate cost functions that capture change and avoid overfitting hazards. For simpler cases, such as low noise and separated nonlinear phenomena as the examples visualized above, supervised learning approaches where an expert demonstrates a few examples of emergent dynamics, the system could potentially search for cost-thresholds that yield appropriate model responses in regards to equation R3, such as a jump in the number of model parameters, to adapt to the emergent feature. The general problem is clearly challenging. For more complex cases, such as greater noise-to-signal ratio in data and non-isolated nonlinear phenomena, it is likely that the same approach may break down. Exploration of these limitations is deferred to future work. A similar approach is suggested regarding the skewness test of equation D2. Likewise, for simple cases such as the data sets modeled above, a design approach can be pursued. Additionally, in problems where stronger assumptions concerning the residual distribution can be made (e.g., Gaussian), skewness testing may be more interpretable and prescriptive than our first problem of designing cost functions that induce model adaptability.

## Conclusion

The additive logistic model is a straightforward method to fit data that has several surges. Owing to the flexibility and elegance of the logistic function at its core, ALM can be seen as a flexible extension and is shown to be able to model causally episodic growth and decay components in a variety of problems. ALM features efficient construct-ability, simple theory, and interpretability and appears useful as a data-driven model approach for general application. It also points to a general approach for surrogate model construction by introducing other nonlinear component functions. We have demonstrated the utility of this approach and observed how it yields straightforward models to problem domains that lack classical models that can perform as well. Further, our approach requires no outside information in relation to the information about changes and novel surges, but can potentially self-identify change-points and tune novel parameters when novel surges arise.

The model can also be used in more fields beyond those cited here. The model can be used not only to predict change-points, but to aid in considering the severity of events. A major goal in volcanology is to predict how severe the next eruption will be^57^^58^. For rainfall, most research doesn’t report the time when the trend changes^59^, something the model is designed to accomplish. Another field that looks at trends and changes in the trends is the study of invasive species^60^. Climate change is also interested in not just the change in the data, but the magnitude of the change^61^. In future work, we plan to consider multidimensional adaptive models (spatial-temporal) in this context. In the broader field of ecology, estimating the amount of change as well as the time of change is a difficult problem^62^. In some disciplines, the causal events inducing changing dynamics are either not directly observable or (for example, in cybersecurity) are strategically withheld from the modeler, such as a hidden population. For such problems, our framework that links observable change to causal event inference could prove useful to evaluate forensically causal conditions or interventions, and thereby offer enhanced analysis modalities to a variety of pressing problems.

## Supplementary Information

Supplementary Information.

## Data Availability

The Datasets used for model development are publicly available from the following detailed sources [For a complete list of sources, including appended materials, see: https://github.com/austincasey/humpty/tree/main/data]. COVID-19 case loads were collected daily (up to mid-2023) on a per-US-county basis and shared via the usafacts.org website61, this was filtered by county and used as input with ALM to form Figures: 1, 3a, b, c, and d. The US Bureau of Reclamation measures Lake Mead monthly to determine how much water was lost or gained and shares that information with the public53. This data was used with our ALM model to generate Figure 2a. The Consumer Price Index (CPI) is tracked by the St. Louis Federal Reserve Bank and is published monthly54. This data was used with ALM to produce Figure 2b. Data on cybersecurity vulnerabilities are collected by Mitre Corporation and made available to the public62. This data is used with ALM to form Figure 2c. COVID-19 social media data was collected from the GDELT63 project. These data were filtered by location (Las Vegas) and used with ALM to form Figure 2d. All source code developed for this research is publicly available at https://github.com/austincasey/humpty along with copies of filtered data sets from above. For any additional questions, the corresponding authors can assist upon reasonable request.
