# Supplementary material for: Modeling and inference of mixed dynamics and detection of causal emergent features
Source: Sci Rep. 2026 Jan 14;16:2228. doi: 10.1038/s41598-025-29523-z (PMC12815911; doi:10.1038/s41598-025-29523-z)
Supplement: Supplementary file 1 — Supplementary Information. [file 41598_2025_29523_MOESM1_ESM.pdf]

# Appended materials

## 5.1 COVID-19 forecasting

To assess effectiveness of our methods for projections of COVID-19 cases, we selected a geographical region (the state of California) during the pandemic time frame (interval from 2020-01-22 through 2023-03-09) to apply direct comparison to other widely known pandemic models which offer predictions. The use of our Affine model with logistic equations, which we term ALM performed well and is competitive with other methods for prediction tasks.

Below we discuss how our baseline model, termed *ALM-baseline*, and how it is formed to predict 28 days (or 4 weeks) of data ahead at each point in time (the ALM model uses only historical data at each time point). With four week predictions we can form direct comparisons to other models whose forecasts are available via the COVID-19 Forecast hub[64]. We continue to describe the mathematics of direct pairwise comparisons to other models and conclude with a summary.

### *5.1.1 The ALM-baseline model for COVID-19 pandemic*

ALM-baseline model for COVID-19 (confirmed cases count) from California over the period 2020-01-22 through 2023-03-09, in order to compare ALM projections and forecasts to those of other methods which furnish forecasts. Notice, to do this, we avoid the aforementioned problem of determining when conditions for which the model should automatically detect its requirements for additional parameters. Rather, for these comparisons, we provide time points for the ALM model to make its jumps from $k$ to $k+1$ active growth modes. These jumps were selected in retrospective analysis, but are fixed to time points when a human in the loop (at the forecast date) would be able to do the same, or at least could be considering various hypotheses. We selected these jump points in a fairly conservative manner to ensure that evidence of novel surge would be clear at the interceding time point. Ideally, an expert who maintains the model on a daily basis would have already compared various hypotheses with hypothesis testing (e.g., a ALM-$k$ vs ALM-$(k+1)$) to determine if/when the addition of new parameters is justifiable within a decision context of other information, such as leading indicators that a novel surge in other regions of the world may anticipate the need to increment the ALM model.

While we have defined the primitive regularization terms which can potentially help to automate a self-aware model fully in our future work. Toward the goal of automating further model intelligence, our future work will focus on:

1. persistence of historical parameters from the baseline, and recent divergence,
2. Variation in Residual fit and skew statistics using historical parameters vs. current parameter estimates.

For this comparison, our jump points are labeled in Table A1 as $J_k$ the time point (days since first collected 2020-01-22) when the model introduces the $k^{\text{th}}$ hump.

| | 1 | 2 | 3 | 4 | 5 | 6 | 7 |
|---|---|---|---|---|---|---|---|
| jump | $J_1$ | $J_2$ | $J_3$ | $J_4$ | $J_5$ | $J_6$ | $J_7$ |
| num params | 3 | 6 | 9 | 12 | 15 | 18 | 21 |
| day | 1 | 182 | 336 | 581 | 735 | 875 | 1050 |

**Table A1.** ALM-baseline

Below we provide our forecasts for two time-points $t = 353$ in Figure A1, and $t = 841$ in Figure A2:

### *5.1.2 Comparison to other methods*

For this comparison, we select to model California COVID-19 confirmed cases during the period from 2020-01-22 through 2023-03-09. Using the reference data from COVID-19 Forecast Hub[64], we compare ours to other methods which offer predictions over some or all of the time period. We selected only methods which offer more than 80 predictions for comparison. These methods are summarized in Table A2 below.

To compare ALM to the published forecasts methods/models, some accommodation to the particulars of each method is needed. Since methods offer various number of predictions at various times (including periodic), there is no fair method to compare all at once. Rather, we can perform a pairwise comparison of ALM to each model, since our ALM-baseline (offering 28 days of prediction for each data point) is complete enough to match forecast predictions of any model's particular dates (so long as the dates are within the interval of collected data).

Letting *method-X* range over the other methods, we will perform a pairwise runoff analysis of forecast accuracy for ALM vs. method-X basis where method-X.

Fixing one of the models as method-X we perform the following steps to obtain a comparison:

1. Create a comparable set of predictions matching method-X directly.

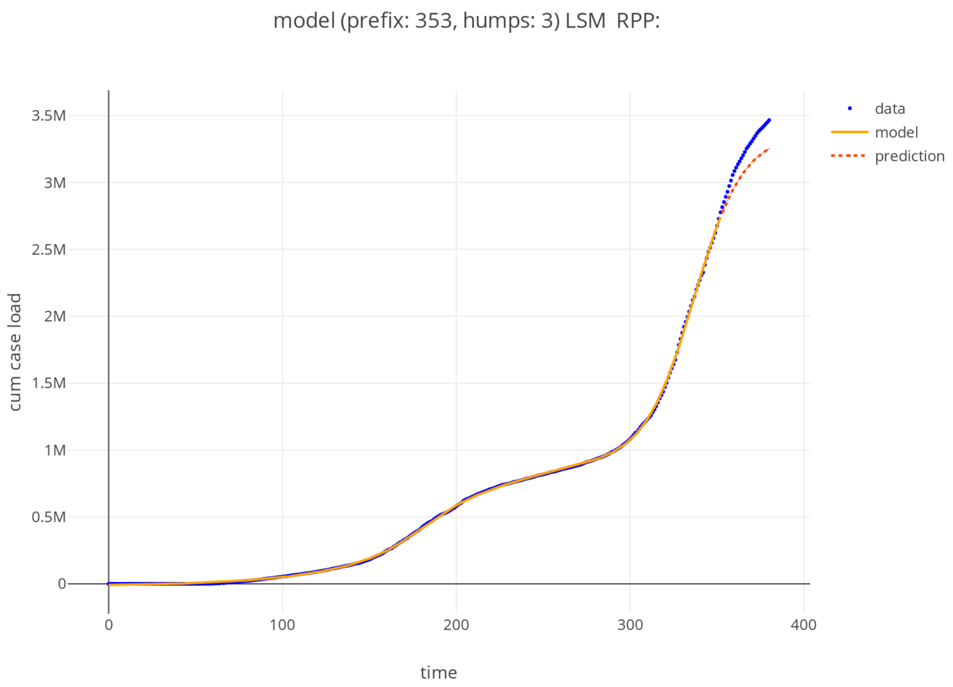


**Figure A1. ALM-baseline forecast viewed at time point** 353 **having** 3 **sets of logistic parameters**. The ALM model uses only data for cumulative confirmed cases up to and including day 353. Note that the model (orange) is hard to distinguish from the data (blue). After day 353 the ALM model prediction (or forecast) is shown as a dotted red line, and extends over 28 additional days (or four weeks). Notice the prediction and data (blue dots) have similar qualitative features. In this case, both the data and prediction are indicative of growth reaching capacity.

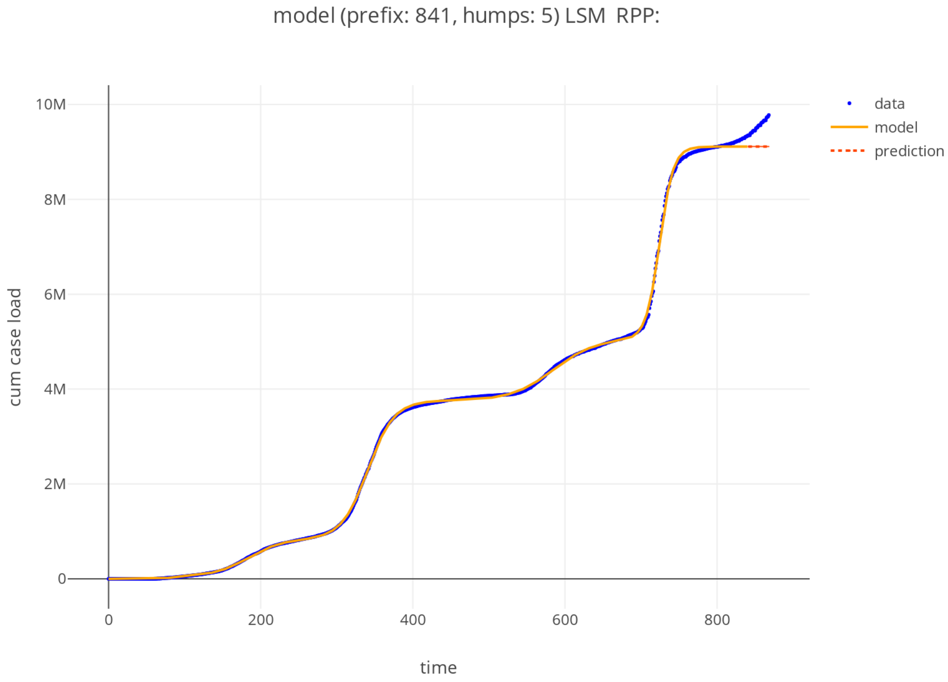


**Figure A2. ALM-baseline forecast at time point** 841 **having** 5 **sets of logistic parameters**. The ALM model uses only data for cumulative confirmed cases up to and including day 841. Note that the model (orange) is hard to distinguish from the data (blue). After day 841 the ALM model prediction (or forecast) is shown as a dotted red line, and extends over 28 additional days (or four weeks). Notice the prediction struggles to anticipate a noticeable increase in cumulative confirmed cases. In this case, additional evidence will eventually confirm the need for another logistic component.

| method | total preds made | preds points total | preds per point (max) |
|---|---|---|---|
| COVIDhub-baseline | 612 | 153 | 4 |
| COVIDhub-4weekensemble | 540 | 135 | 4 |
| CU-select | 532 | 133 | 4 |
| BPagano-RtDriven | 492 | 123 | 4 |
| JHUIDD-CovidSP | 475 | 119 | 4 |
| RobertWalraven-ESG | 468 | 117 | 4 |
| USC-SIkJalpha | 460 | 115 | 4 |
| JHUAPL-Bucky | 420 | 105 | 4 |
| Karlen-pypm | 412 | 103 | 4 |
| CovidAnalytics-DELPHI | 412 | 103 | 4 |
| Microsoft-DeepSTIA | 395 | 99 | 4 |
| UVA-Ensemble | 384 | 96 | 4 |
| COVIDhub-trainedensemble | 384 | 96 | 4 |
| CEID-Walk | 373 | 94 | 4 |
| MUNI-ARIMA | 348 | 90 | 4 |
| UMich-RidgeTfReg | 336 | 84 | 4 |
| MITISOLAT-Mixtures | 332 | 83 | 4 |
| MIT-Cassandra | 332 | 83 | 4 |
| KITmetricslab-select_ensemble | 332 | 83 | 4 |
| COVIDhub-ensemble | 315 | 135 | 4 |
| COVIDhubCDC-ensemble | 290 | 75 | 4 |
| IEMMED-CovidProject | 272 | 68 | 4 |
| JHUCSSE-DECOM | 269 | 68 | 4 |
| UCLA-SuEIR | 268 | 67 | 4 |
| LANL-GrowthRate | 260 | 65 | 4 |
| JHUUNCGAS-StatMechPool | 224 | 56 | 4 |
| Covid19Sim-Simulator | 224 | 56 | 4 |
| LNQ-ens1 | 192 | 48 | 4 |
| MOBS-GLEAMCOVID | 168 | 42 | 4 |
| IowaStateLW-STEM | 168 | 42 | 4 |
| DDS-NBDS | 140 | 35 | 4 |
| LosAlamosNAU-CModelSDVaxVar | 120 | 30 | 4 |
| LUcompUncertLab-VAR3streams | 120 | 30 | 4 |
| PRUMD-CFRepTiLe | 88 | 22 | 4 |
| IHME-CurveFit | 88 | 22 | 4 |
| AMM-EpiInvert | 84 | 21 | 4 |

**Table A2.** COVID-19 forecast models that are selected for comparison to ALM.

2. Calculate a *MSE sequence* for each method over the comparable prediction time points.

3. Calculate a total MSE score over the comparable prediction time points

**Step 1, comparable set of predictions:** For each date $d$ when method-X offers a forecast (future prediction) we record the date of forecast and the 'target-dates' $t_d = [t_1, t_2, \cdots, t_k]$ for which method-X furnishes predictions. We label the dates for which method-X offers predictions as: $D = [d_1, d_2, \cdots, d_M]$. Note if multiple predictions are provided by method-X we filter the forecast (signal is *confirmed incidence num*, quantile is 0.5) as the most relevant forecast for comparison.

**Step 2, calculate a MSE sequence:** For each date $d \in D$, let target dates $t_d = [t_1, t_2, \cdots, t_k]$ be the time points at which method-X furnishes predictions using only data up to date $d$. We do likewise, using data up to and including date $d$ but no further, the ALM model (fitted to data historical to and including date $d$) is then used to predict *confirmed incidence num* at forward times $t_1, t_2, \cdots, t_k$ matching predictions of model-X. We label the forecast counts of method-X as $V_{d,1}, V_{d,2}, \cdots, V_{d,k}$ and the forecast counts of ALM as $W_{d,1}, W_{d,2}, \cdots, W_{d,k}$. Since our dataset of *confirmed incidence num* only includes data from 2020-01-22 to 2023-03-09 inclusive, we limit the comparisons to only include dates and target-dates within the data collection interval $I = [2020\text{-}01\text{-}22, 2023\text{-}03\text{-}09]$ We are able to derive ALM forecasts for any $d \in I \cap D(X)$. Further, we are able to compare ALM forecasts with that of method-X so long as $(d \in I \cap D(X)) \wedge \left( \bigwedge_{t_j \in t_d} t_j \in I \cap D(X) \right)$.

Letting $C_i$ be the confirmed incidence number for each $i \in I$ be actual counts from data, letting $T = D(X) \cup I$, we can calculate a *forecasting-MSE* function for both method-X and ALM as follows:

$$\text{MSE}_X : T \to \mathbb{R} : i \to \left( \sum_{j \in t_i} \sqrt{(C_j - V_{i,j})^2} \right)$$

while

$$\text{MSE}_\text{h} : T \to \mathbb{R} : i \to \left( \sum_{j \in t_i} \sqrt{(C_j - W_{i,j})^2} \right)$$

Both functions are defined for $i \in T = D(X) \cup I$, the plot of which indicates the forecast accuracy as a function of date.

The forecast accuracy functions are plotted below in Figures A4, A5, and A6.

**Step 3, Calculating and comparing total MSE scores:** Finally, a view of all pairs method-X vs. ALM is provided by calculating the total forecast accuracy comparison point as:

$$(x, y) \;\text{ with }\; x = \sum_{i \in T} MSE_X(i) \;\text{ and }\; y = \sum_{i \in T} MSE_h(i)$$

These points are plotted in log-log scale below in Figure A3. Note that the line $x = y$ represents *equal quality outcomes* for overall forecast error. Points $x > y$ represent outcomes where the forecast quality (measured as sum MSE) of ALM is better than that of method-X.

In Figure A4 we compare to Delphi[65], a *Now-casting* method is based on estimation of time delay distribution and deconvolution of a joint distribution of symptom onset to case count.

#### 5.1.3 Summary

The ALM-baseline provides reliable forecasts competitive with other models from COVID-19 Forecast Hub that offer more than 80 predictions over the given interval within the state of California during the pandemic. As our model, with such few assumptions, can provide insights for other mathematical modelers working to improve forecasts needed by policymakers and pandemic planners. The performance of ALM relies on two aspects: 1) multiple relevant nonlinear models, and 2) the ability to remix those models in mixture form. Other models such as Delphi and the Los Alamos model further help us see both the opportunities and challenges of our proposed method. We speculate that the curvature of the logistic function is to credit for time periods where ALM MSE improves upon other forecasting methods, for example during a surge once enough data can be leveraged to estimate the capacity. By similar reasoning, the parameters of growth and capacity tend to be overestimated during the early stages of a surge, resulting in periods where ALM struggles to regulate forecast MSE compared to other models. Views of both benefit of curvature and struggle can be seen with separation of MSE sequences in Figures A1 and A2 respectively. One possibility, that we consider, are various regularization concepts to address forecasting issues. We defer this question for future work.

### 5.2 Mathematical model

The dynamics of equation M1 can be approximated with simpler forms in two important end cases. Throughout the discussion, we will focus on the growth regime $r > 0$ and $X(t) << K$, and in general, a similar intuition can be found for the decay regime when $r < 0$ and $X(t) >> K$.

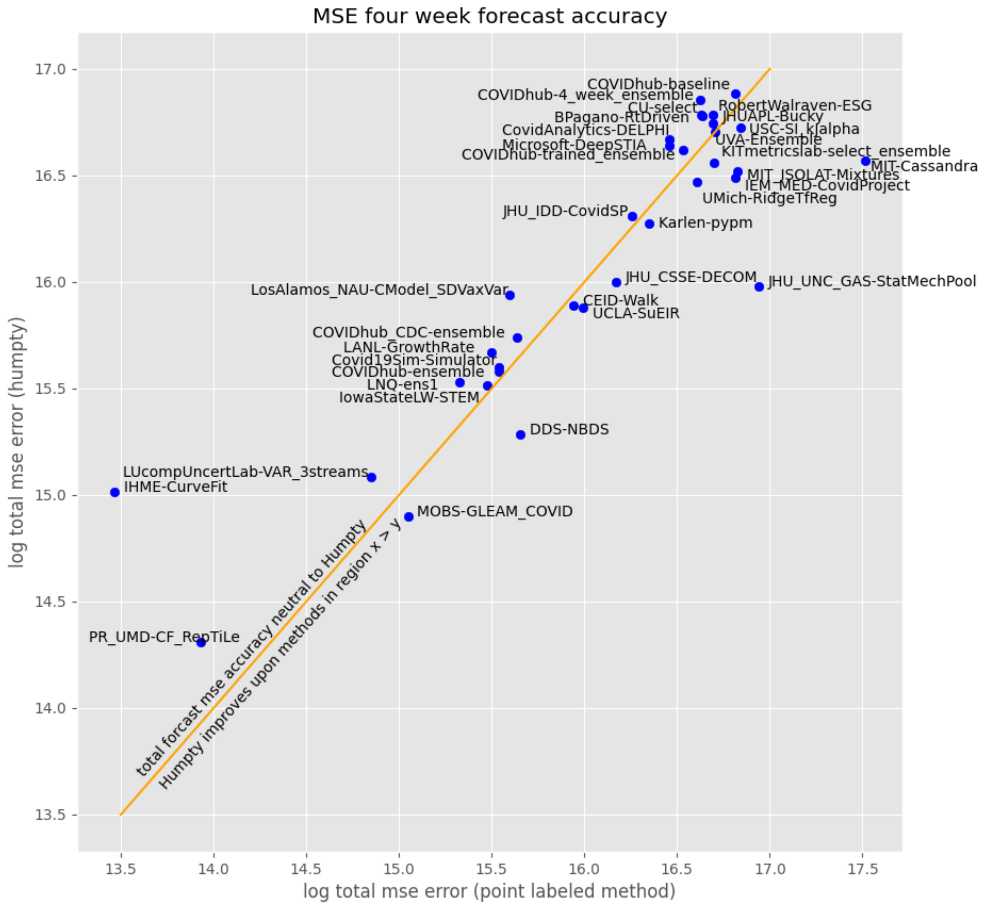


**Figure A3.** **Forecasting error comparison of ALM (humpty) vs. methods with forecasts posted on COVID-19 forecast hub**. The point locations indicate a direct comparison of total overall MSE for forecasts. The x-axis shows the MSE for the labeled method, while the y-axis shows the MSE for the ALM-baseline. Points below the diagonal indicate that the ALM model performs better at forecasting tasks.

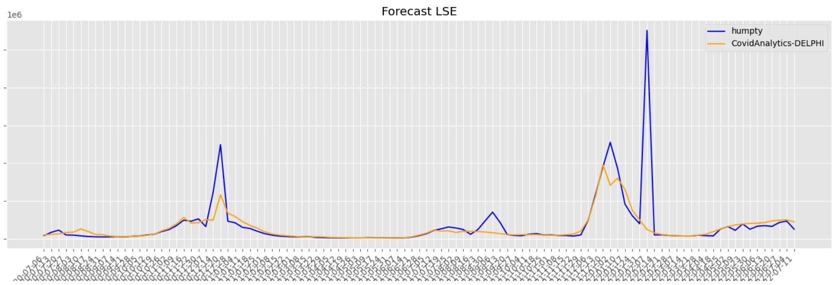


**Figure A4.** **ALM (humpty) and Delphi MSE sequences**: Each method Delphi and ALM produce forecasts for confirmed case counts, ALM matches the number of predictions by drawing from its baseline model. The MSE for forecast error is illustrated as a function of the time when predictions are made.

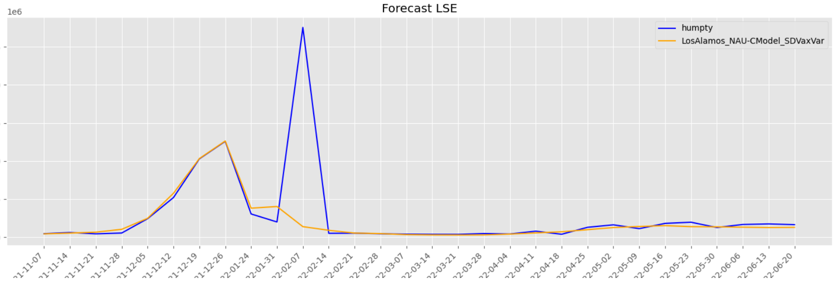


**Figure A5. ALM (humpty) and Los Alamos MSE sequences**. Each method, Los Alamos' and ALM , produce forecasts for confirmed case counts, ALM matches the number of predictions by drawing from its baseline model. The MSE for forecast error is illustrated as a function of the time when predictions are made.

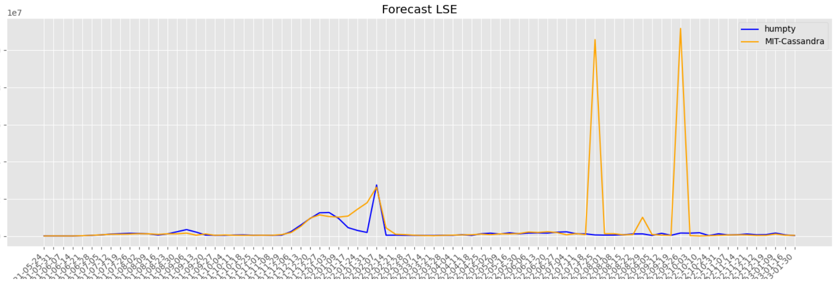


**Figure A6. ALM (humpty) vs MIT Cassandra MSE sequences**. MIT's Cassandra and ALM produce forecasts for confirmed case counts. ALM matches the number of predictions by drawing from its baseline model. The MSE for forecast error is illustrated as a function of the time when predictions are made.

In the first case, when $X(t) << K$, the term $(1 - \frac{X(t)}{K}) = 1 - \varepsilon \approx 1$ reveals exponential growth driving the dynamic increase of $X(t)$ upward with only a small resistance of $\varepsilon$, Given the initial conditions, this implies that for $t$ close to $t_0$, the growth trajectory is close to that of exponential. Second Case: As $X(t) \to K$ the term $(1 - \frac{X(t)}{K}) \to 0$ freezes growth / decay as $t \to \infty$, thus inducing $X(t)$ toward the equilibrium of $K$.

Further, from the theory of the qualitative study of the ODE, the solution can be concluded to be continuous and non-decreasing, thereby implying that the first case of exponential growth will eventually transition to the second case of a constant (e.g., frozen) function.

We can go further; the solution (analytic) in S1 can be shown to be infinitely differentiable as a rational expression of polynomials over exponential functions (also infinitely differentiable). See Figure A7. Therefore, we can calculate directly the

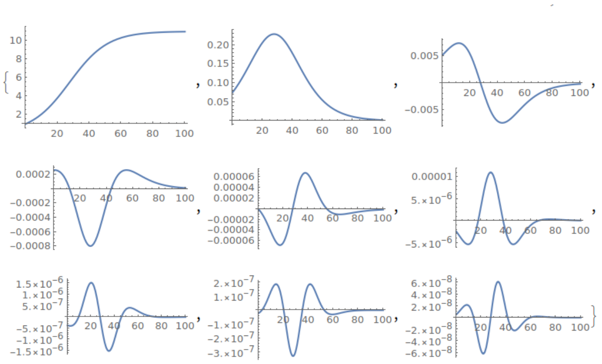

**Figure A7. Logistic growth solution and higher order derivatives**.

inflection point where the second variation of the function is zero, by using the analytical solution of S1.

#### 5.2.1 The inflection point:

Letting

$$X(t) = \frac{JK}{J + (K-J)e^{-rt}}, \tag{X1}$$

we match S1 when $J = X_0$. Carrying out differentiation with respect to $t$ yields:

$$\frac{d}{dt}X(t) = \frac{e^{rt}J(K-J)Kr}{((e^{rt}-1)J+K)^2} \tag{1}$$

$$\frac{d^2}{d^2t}X(t) = \frac{e^{rt}J(J-K)(J+e^{rt}J-K)Kr^2}{((-1+e^{rt})J+K)^3}. \tag{2}$$

Solving $\frac{d^2}{d^2t}X(t) = 0$ for $t$, we obtain, as our first derivation, a solution for the logistic inflection point as a closed bounded formula in $J, K$ and $r$:

$$\tau(J,K,r) = \frac{\log \frac{K-J}{J}}{r}. \tag{D1}$$

One can check that for $t = \tau(J,K,r)$ in equation X1, the inflection point:

$$(\tau, \frac{K}{2}),$$

can be confirmed to be a point on the solution curve X1. Recall that this is an important point, reflecting a momentary balance of growth and resistance.

### 5.3 Relation of the logistic ODE and the hyperbolic tangent (tanh) function

Starting from the identity:

$$\tanh(x) = \frac{e^x - e^{-x}}{e^x + e^{-x}},$$

we can obtain the identity:

$$\frac{1+\tanh(x)}{2} = \frac{1}{1+e^{-2x}}. \tag{B1}$$

Equivalent versions of the logistic curve S1, are obtained by first dividing by $X_0$, and then by rewriting exponents as:

$$\frac{K}{\left(\frac{K-X_0}{X_0}\right)e^{-rs}+1} = \frac{K}{e^{-rs+\log\left(\frac{K-X_0}{X_0}\right)}+1}.$$

Letting $\omega = \frac{r}{2}\left(s - \frac{\log\left(\frac{K-X_0}{X_0}\right)}{r}\right)$, and $\kappa = \frac{K}{2}$, the logistic curve can now be re-written in hyperbolic tangent form with B1:

$$K\frac{1}{1+e^{-2\omega}} = \kappa\left(1+\tanh(\omega)\right).$$

Finally, letting $s = t - t_0$, we can alternatively represent:

$$\frac{K}{\left(\frac{K-X_0}{X_0}\right)e^{-r(t-t_0)}+1} = \kappa\left(1+\tanh(\alpha t+\beta)\right), \tag{B2}$$

with:

$$\kappa = \frac{K}{2}, \alpha = \frac{r}{2}, \beta = -\frac{r}{2}\left(t_0 + \frac{\log\left(\frac{K-X_0}{X_0}\right)}{r}\right).$$

Letting:

$$g(t|\kappa,\alpha,\beta) = \kappa\left(1+\tanh(\alpha t+\beta)\right),$$

the gradient $\nabla g$ (with respect to parameters) can be calculated as:

$$\begin{aligned}
\frac{\delta g}{\delta \kappa} &= (1+\tanh(\alpha t+\beta)) \\
\frac{\delta g}{\delta \alpha} &= \kappa t \operatorname{sech}^2(\alpha t+\beta) \\
\frac{\delta g}{\delta \beta} &= \kappa \operatorname{sech}^2(\alpha t+\beta).
\end{aligned}$$

### 5.4 Mixture model

Functions of the form:

$$\sum_{i=1}^{N} f(t;\phi_i)$$

can be alternatively expressed as:

$$\sum_{i=1}^{N} g(t;\theta_i) = \sum_{i=1}^{N} \kappa_i\left(1+\tanh(\alpha_i t+\beta_i)\right),$$

with $\theta_i = \langle \kappa_i, \alpha_i, \beta_i \rangle$, the last form showing a form that is well suited for projection methods.

### 5.5 Prediction of regions of change

The close connection between the logistic curve and the hyperbolic tangent function affords a means to obtain closed bounded integral formulae. One application is that the temporal region where a percent of change due to the effects of a single cause (i.e., logistic parameters $J, K, r$) can be determined.

Using equation B2, $\tanh(\alpha t+\beta)$ can be seen to have a zero at $\tau = t_0 + \frac{\log\left(\frac{K-X_0}{X_0}\right)}{r}$. We can also use the formula:

$$\tanh^{-1}(p) = \frac{1}{2}\log\frac{1+p}{1-p}, \tag{D1}$$

to derive the following inverse formula. Letting $q$ be a fraction of total displacement $K$:

$$g(t|\kappa,\alpha,\beta) = qK,$$

for $q \in [0,1]$. Solving for $\omega$, or time when:

$$\frac{K}{2}\left(1+\tanh(\omega)\right) = qK,$$

can be simplified to $\omega$ so that:

$$\tanh(\omega) = 2q-1.$$

Using equation D1, we can show:

$$\omega = \frac{1}{2}\log\frac{q}{(1-q)},$$

This occurs when:

$$(t-\tau) = \frac{1}{r}\log\frac{q}{(1-q)}.$$

We summarize the result:

$$f(t;r,K,t_0,X_0) = qK \text{ when } t = \tau + \frac{1}{r}\log\frac{q}{(1-q)} \tag{Lemma 1}$$

The inverse formula of Lemma 1 provides a tool to bound the motion due to growth components governed by parameters $(K, r, t_0, X_0)$.

For example, the inference problem may be able to estimate parameters $K, r, t_0, X_0$ even before time observation of data at the inflection point $\tau$, nonetheless Lemma 1 can be used as early as estimates become stable, enabling the prediction of a future time point when 80% of the growth will be experienced in the data.

We summarize useful formulae for the logistic curve, and indicate the derivation and utility as tools in algorithms.

Given a growth hypothesis parameterized by$K, r, t_0, X_0$:

- Derived with D1, Determine the inflection point $\tau$.
- Determine the symmetric interval about $\tau$ with $p$ percent change: Derived with Lemma 1, let $\beta = \log\left(\frac{1-\frac{p}{2}}{\frac{p}{2}}\right)$, the symmetric interval $[\tau - \frac{\beta}{r}, \tau + \frac{\beta}{r}]$ will contain growth from $\left(1-\frac{p}{2}\right)K$ to $\frac{p}{2}K$.
- Determine the time point at which $q$ of the change (due to $K, r, t_0, X_0$) will be observed as historical (for $q < \frac{1}{2}$): Derived with Lemma 1 as $t = \tau + \frac{1}{r}\log\left(\frac{q}{1-q}\right)$.

### 5.6 Numerical least squares optimization

To overcome hazards such as slow convergence or convergence to local minima in the nonlinear least squares procedure, we implement a set of heuristics. The model is created in an iterative way based on the number of addends, from 1 to 15. The first step is the initial conditions that the program uses. We begin by using a change-point method to approximate where the model could be changing, that is, one logistic function ends and another begins. The binary segmentation[66] method is used. We use this because it takes as a parameter the number of potential change-points expected and returns an educated guess based on the data. The implementation[67] we use is efficient and facilitates quick convergence. The initial values for the midpoints of the fit and the capacity are derived directly from the data.

At each step, the best fit was found and the second result was compared with the original data. This was done by considering the difference between the expected value and the model value for each data point, squaring it, and adding it to a sum. The sum of squares was then computed, that is, the difference between the expected value and the mean of the expected value. Finally, the R-squared value was calculated by dividing the first value by the second and subtracting that from 1.0. If the value of the R-square was between 0.997 and 1, then that curve is considered the best fit. Otherwise, the algorithm continues until all the sums are computed and returns the curve with the R-Squared value closest to 1.

### 5.7 Equivalence of logistic and hyperbolic tangent forms from Taylor series expansions.

Expanding Taylor series about $x = 0$, for $\tanh(x)$, and $\frac{1-e^{-rx}}{1+e^{-rx}}$, we obtain:

$$
\begin{aligned}
\tanh(x) =& x - \frac{x^3}{3} + \frac{2x^5}{15} - \frac{17x^7}{315} + \frac{62x^9}{2835} - \frac{1382x^{11}}{155925} + \\
& \frac{21844x^{13}}{6081075} - \frac{929569x^{15}}{638512875} + \frac{6404582x^{17}}{10854718875} \\
& - \frac{443861162x^{19}}{1856156927625} + O\left(x^{20}\right), \text{and} \\
\frac{1-e^{-rx}}{1+e^{-rx}} =& \frac{rx}{2} - \frac{r^3x^3}{24} + \frac{r^5x^5}{240} - \frac{17r^7x^7}{40320} \\
& + \frac{31r^9x^9}{725760} - \frac{691r^{11}x^{11}}{159667200} + \frac{5461r^{13}x^{13}}{12454041600} \\
& - \frac{929569r^{15}x^{15}}{20922789888000} + \frac{3202291r^{17}x^{17}}{711374856192000} \\
& - \frac{221930581r^{19}x^{19}}{486580401635328000} + O\left(x^{20}\right).
\end{aligned}
$$

Which can be shown as an identical expansion when $r = 2$.

### 5.8 Measuring the discrepancy between an ODEs initial condition problem and the alternate data inference problems

By including a slight variant of the model above with an indicator function multiplier, it is possible to identically model a set of ODEs with various initial conditions as:

$$\sum_{i=1}^{N} X_i(t) = \sum_{i=1}^{N} f(t;\phi_i)\mathbb{1}_{[t_{i0},\infty)}.$$

With $X_i(t)$ a solution to ODE as equation S1, with parameters $(r_i, K_i)$ and initial conditions $(t_{i0}, X_{i0})$.

Let,

$$f(t;r,K,t_0,X_0) = \frac{X_0 K}{(K - X_0)\, e^{-r(t-t_0)} + X_0}. \tag{$\mathscr{M}_1$}$$

Note the relation: $X(t) = f(t;r,K,t_0,X_0)\mathbb{1}_{[t_0,\infty)]}$, where the indicator function is defined as:

$$\mathbb{1}_{[t_0,\infty)}(t) = \begin{cases} 1 \text{ when } t > t_0 \\ 0 \text{ o.w.} \end{cases}.$$

The solution of the Ordinary Equation $X(t)$, only differs from $f(t;r,K,t_0,X_0)$ for $t < t_0$, the discrepancy given in integral form as:

$$\int_{-\infty}^{\infty} |X(t) - f(t;r,K,t_0,X_0)|dt = \int_{-\infty}^{t_0} f(t;r,K,t_0,X_0)dt.$$

This can also be calculated centering time to the inflection point and determining a crossing time (see equation S4) $t_q$ for $q = X_0/K$. Since initial conditions usually precede the vast majority of dynamic change, the discrepancy can generally be reasoned to be small in such cases.

### 5.9 Modes of logistic equations, and parameter constraints

Let $\mathscr{M}$ be the class of curves in equation ($\mathscr{M}_1$), ranging over parameter space $t_0 \in (-\infty,\infty)$ and $r, K, X_0$ such that:

$$\mathscr{P} = \{r, K, X_0 \in \mathbb{R}^3 : r(K - X_0) \in [0,\infty)\}. \tag{$\mathscr{M}_S$}$$

Note that this condition is enough to include the case of growth, decay and constant functions. We say that a point in the parameter space is admissible if it is contained in $\mathscr{P}$, that is, it satisfies the condition of $\mathscr{M}_S$.

## Source code

All source code developed for this research is publicly available: https://github.com/austincasey/humpty

Within the source repository, we provide copies of datasets used in this study.

## Datasets used in the appendix

The Datasets used in this appendix are publicly available from the following detailed sources. COVID-19 case loads were collected daily (up to mid-2023) on a per-US-county basis and shared via the usafacts.org website[61], this was filtered by county and used as input with ALM to form Figures: A1, and A2. CovidHub[64] which aggregated COVID19 data, along with the many forecast methods, and makes that data publically available at: https://covid19forecasthub.org/. We used this data along with ALM to form Table: A2 and Figures: A3, A4, A5, and A6.
